# Novel Method of Detecting Movement of the Interference Fringes Using One-Dimensional PSD

**DOI:** 10.3390/s150612857

**Published:** 2015-06-02

**Authors:** Qi Wang, Ji Xia, Xu Liu, Yong Zhao

**Affiliations:** 1 College of Information Science and Engineering, Northeastern University, Shenyang 110819, China; E-Mails: neuliuxu@gmail.com (X.L.); zhaoyong@ise.neu.edu.cn (Y.Z.); 2 State Key Laboratory of Synthetical Automation for Process Industries, Northeastern University, Shenyang 110819, China; 3 Academy of Ocean Science and Engineering, National University of Defense Technology, College of Optoelectronic Science and Engineering, Changsha 410073, China; E-Mail: blovexiaji@163.com

**Keywords:** fiber M-Z interferometer, centroid detection, movement measurement, PSD

## Abstract

In this paper, a method of using a one-dimensional position-sensitive detector (PSD) by replacing charge-coupled device (CCD) to measure the movement of the interference fringes is presented first, and its feasibility is demonstrated through an experimental setup based on the principle of centroid detection. Firstly, the centroid position of the interference fringes in a fiber Mach-Zehnder (M-Z) interferometer is solved in theory, showing it has a higher resolution and sensitivity. According to the physical characteristics and principles of PSD, a simulation of the interference fringe’s phase difference in fiber M-Z interferometers and PSD output is carried out. Comparing the simulation results with the relationship between phase differences and centroid positions in fiber M-Z interferometers, the conclusion that the output of interference fringes by PSD is still the centroid position is obtained. Based on massive measurements, the best resolution of the system is achieved with 5.15, 625 μm. Finally, the detection system is evaluated through setup error analysis and an ultra-narrow-band filter structure. The filter structure is configured with a one-dimensional photonic crystal containing positive and negative refraction material, which can eliminate background light in the PSD detection experiment. This detection system has a simple structure, good stability, high precision and easily performs remote measurements, which makes it potentially useful in material small deformation tests, refractivity measurements of optical media and optical wave front detection.

## 1. Introduction

Laser interferometry, with its advantages of large measuring range, high resolution and accuracy, *etc.*, has been widely used in the field of precision and ultra-precision measurement [1]. Numerous experiments have established that the measurement of interference fringe movement is one of the most important components in the measurement system. Traditional measurement of interference fringes compares the images of interference fringes sampled by CCD under different states, but it is limited by the CCD device’s size and large array, which makes the detection system expensive and requires a complicated ancillary circuit [2]. With the development of semiconductor optoelectronic technology, PSD has shown more obvious advantages than CCD in terms of performance and price, and there is a growing trend to use PSD instead of CCD for interference fringe movement detection [3]. Research on PSD technology was a hot issue in the measurement field at the end of the 20th century [4,5,6]. It is mainly applied in industrial detection and monitoring [7], automatic focusing [8,9], three-dimensional shape measurement [10], robot sensors [11], laser beam deflection detection technology [12] and laser displacement detection research [13]. Donati *et al.* [14] analyzed the performance of PSD and of distance- or displacement-measuring instruments in 2007. The uncertainty of position-sensing photodiodes was derived first at the quantum limit, and then, it was extended to the more realistic case of the intervening thermal noise or dark current. PSD in our experiment is also disturbed by the background light, which often affects its accuracy and reliability. Therefore, a kind of structure is firstly proposed to fundamentally solve the background light influence on PSDs.

Interferometers have widespread applications in the detection field, and interferometer fringes are tracks of the interference field with the same optical path differences (OPD). Some useful information about OPD can be extracted according to the shape, direction, density and movement of the interference fringes [15,16,17]. As the light splitting and interference in the fiber optic Sagnac and Michelson interferometers are done in the same coupler, this kind of structure is not good for PSD detection of interference fringes. However, the fiber-optic M-Z interferometer can remove the last coupler, so that the interference fringes of two beams generated in the air can be detected by PSD. Therefore, a fiber optic M-Z interferometer is more suitable for the work described in this paper.

In recent years, extensive research on interference fringe measurements has been carried out. These studies can be generally divided into two categories: fringe centerline methods and full grayscale methods. Fringe centerline methods pretreat interference fringes by making gray level corrections, denoising, smoothing, and binarization. Then, the fringe centerline can be extracted [18]. The variation of the centerline can be measured under different curvatures, locations, *etc.*, and the physical variable can be inverted by establishing numerical relationships. Morimoto *et al.* [19] designed an experimental device based on CCD for displacement measurement in 2007. After preprocessing interference fringes collected by CCD, the centerline of the interference fringes can be extracted, and the tiny displacement of piezoelectric ceramic (PTZ) reflecting face can be obtained by the position of the interference fringes centerline. The system is able to achieve a displacement of positioning accuracy of 0.41 nm. The full grayscale method samples a specific position in interference fringe patterns, checks its phase change and analyzes numerical relationships between physical variables and phase changes, and the physical variables can be inverted. Compared with the fringe centerline method, the full grayscale method makes full use of all the gray information in interference fringes, while the fringe centerline method uses only part of the gray information in interference fringes. The fringe centerline method has more advantages in detection speed, but has less location precision than the full grayscale method [20]. Both methods have to collect interference fringes by using CCD or Complementary Metal-Oxide-Semiconductor (CMOS) devices, which greatly increases the cost and complexity of the detection system. This paper proposes a novel method for interference fringe centroid detection based on the full grayscale method, which has a simple algorithm, and avoids the complex image processing by taking PSD as a detection device with low cost and a stable structure.

## 2. Theoretical Analysis and Simulation

As mentioned above, a higher sensitivity can be obtained by detecting the interference fringe movements. Taking the interference fringes centerline method’s algorithm complexity and high cost of the system into account, we propose the interference fringe centroid detection method. The principle of the interference fringes centroid is described as follows: when the external physical variable changes, the interference fringes move as interferometric phase changes, which can be converted into light intensity centroid position changes by the interference measurement. Then, the measured parameters can be derived from the centroid position. In measurement technology, the centroid principle is usually applied to remote object tracking and laser beam deflection measurements. This paper regards the intensity distribution of the interference fringe light as a curve with a certain mass, where each point on the light intensity curve equals the quality of the curve. Finally, we can conclude the interference fringe characteristics from the interference fringe intensity centroid detection.

When the interference fringes from a M-Z interferometer are displayed on the viewing screen vertically, the light intensity distribution in the axial direction is described by Equation (1):

(1) $$I(x)=1+\text{γ}\cos(\frac{2N\text{π}x}{\Delta }+\text{δ})$$

where γ is the contrast of the interference fringes, δ is the interference fringes phase difference, Δ is the length of the viewing screen and *N* is the cycles of interference fringes within the viewing screen. Then, based on the centroid principle, the light intensity centroid of the one-dimensional interferogram $\bar{\text{X}}$ is given by Equation (2):

(2) $$\bar{X}=\frac{\int_{-\Delta /2}^{\Delta /2}xI(x)\text{d}x}{\int_{-\Delta /2}^{\Delta /2}I(x)\text{d}x}$$

By combination of Equations (1) and (2), the light intensity centroid of the one-dimensional interferogram $\bar{\text{X}}$ behaves as Equation (3):

(3) $$\bar{X}=\frac{\text{γ}\Delta \sin\text{δ}[\frac{\cos(\text{π}N)}{\text{π}N}-\frac{\sin(\text{π}N)}{{\left(\text{π}N\right)}^{2}}]}{2[1+\text{γ}\cos\text{δ}\frac{\sin(\text{π}N)}{\text{π}N}]}$$

**Figure 1:**
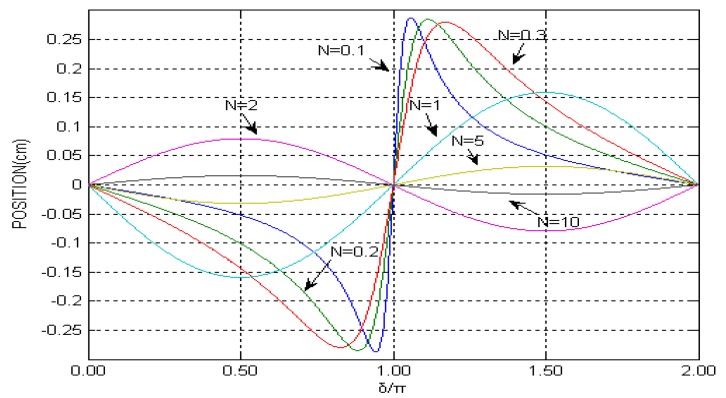
Centroid position *versus* interference fringes phase difference for some values of N.

Within the range of the viewing screen, the number of interference fringes N is always a multiple of the interference cycle and doesn’t change with the initial phase. Figure 1 is the variation of the centroid position of the interference fringes at different values of N, which indicates that the phase difference movement varies periodically with the increase of N. Obviously, the response curve is more sensitive at δ = π, and the same phenomenon also appears at δ = 0 and 2π, which behaves significantly as the N decreases.

**Figure 2:**
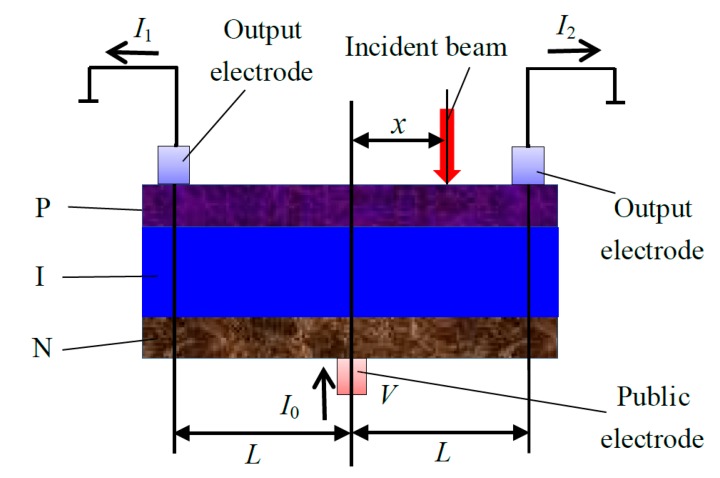
The structure of a Position Sensitive Detector.

PSD is an optoelectronic device, based on the lateral photoelectric effect of an inhomogeneous semiconductor, which is sensitive to the position of the incident light or particle. The schematic of a PSD is shown in Figure 2. When the incident light spot irradiates a PSD photosensitive surface, due to lateral photoelectric effects, and a transverse electric potential exists between the incident point and signal electrode. The photocurrent is divided in the diffusion layer, and the current through the electrodes at both ends are *I*_1_ and *I*_2_, respectively.

The intensities of *I*_1_ and *I*_2_ are determined by the equivalent resistance between the incident point position and the two electrodes. According to the current output, we can detect the centroid position of incident light directly. The formula is as follows:

(4) $$x=L\cdot (I_{2}-I_{1})/(I_{1}+I_{2})$$

where, *L* is the distance between the PSD photosensitive surface midpoint and signal electrodes, and *x* is the distance between the incident light spot point and PSD midpoint. According to the transverse photoelectric effect equation (Lucovsky equation [21]), the instantaneous current flowing from the two PSD electrodes is described by Equations (5) and (6), respectively:

(5) $$I_{1}=\frac{2I_{0}}{\text{π}}\overset{\infty }{\underset{n=1}{\sum }}\frac{1}{n}\sin\frac{\text{π}nx}{2L}\left\{1-\exp\left(\frac{-n^{2}{\text{π}}^{2}t}{4rcL^{2}}\right)\right\}$$

(6) $$I_{2}=\frac{-2I_{0}}{\text{π}}\overset{\infty }{\underset{n=1}{\sum }}\frac{\cos n\text{π}}{n}\sin\frac{\text{π}nx}{2L}\left\{1-\exp\left(\frac{-n^{2}{\text{π}}^{2}t}{4rcL^{2}}\right)\right\}$$

where, *I*_0_ is the photocurrent density, *r* is the sheet resistance in the P zone, *c* is the P-N section capacitance per unit area.

When the signal light incident on the PSD photosensitive surface consists of N discrete interference fringes, then, the interference fringes are composed by a small spot of nonhomogeneous light intensity, *I*_0m_ is the light intensity of the mth spot, and the corresponding incident position of which is X_m_, Equations (5) and (6) can be transformed into Equations (7) and (8) according to the linear superposition principle:

(7) $$I_{1}=\frac{2}{\text{π}}\overset{\infty }{\underset{n=1}{\sum }}\frac{1}{\text{n}}\left[\overset{N}{\underset{m=1}{\sum }}I_{0m}\sin\frac{\text{π}nX_{m}}{2L}\right]\left\{1-exp\left(\frac{-n^{2}{\text{π}}^{2}t}{4rcL^{2}}\right)\right\}$$

(8) $$I_{2}=\frac{-2}{\pi }\overset{\infty }{\underset{n=1}{\sum }}\frac{\cos n\text{π}}{n}\left[\overset{N}{\underset{m=1}{\sum }}I_{0m}\sin\frac{\text{π}nX_{m}}{2L}\right]\left\{1-\exp\left(\frac{-n^{2}{\text{π}}^{2}t}{4rcL^{2}}\right)\right\}$$

When the *t* in the Equations (7) and (8) approaches ∞, then, the steady-state currents output from the two PSD electrodes are obtained as follows:

(9) $$I_{1\infty }=\frac{2}{\text{π}}\overset{\infty }{\underset{n=1}{\sum }}\frac{1}{n}\left[\overset{N}{\underset{m=1}{\sum }}I_{0m}\sin\frac{\text{π}nX_{m}}{2L}\right]=\left(\overset{N}{\underset{m=1}{\sum }}I_{0m}\right)\left\{1-\frac{\sum_{m=1}^{N}I_{0m}X_{m}}{2L\sum_{m=1}^{N}I_{0m}}\right\}$$

(10) $$I_{2\infty }=\frac{-2}{\text{π}}\overset{\infty }{\underset{n=1}{\sum }}\frac{\cos n\text{π}}{n}\left[\overset{N}{\underset{m=1}{\sum }}I_{0m}\sin\frac{\text{π}nX_{m}}{2L}\right]=\left(\overset{N}{\underset{m=1}{\sum }}I_{0m}\right)\left\{\frac{\sum_{m=1}^{N}I_{0m}X_{m}}{2L\sum_{m=1}^{N}I_{0m}}\right\}$$

Further, a solution of Equation (9), Equation (10) in Equation (1) can be obtained as:

(11) $$\bar{X}=\frac{\sum_{m=1}^{N}I_{0m}x_{m}-L\sum_{m=1}^{N}I_{0m}}{\sum_{m=1}^{N}I_{0m}}$$

**Figure 3:**
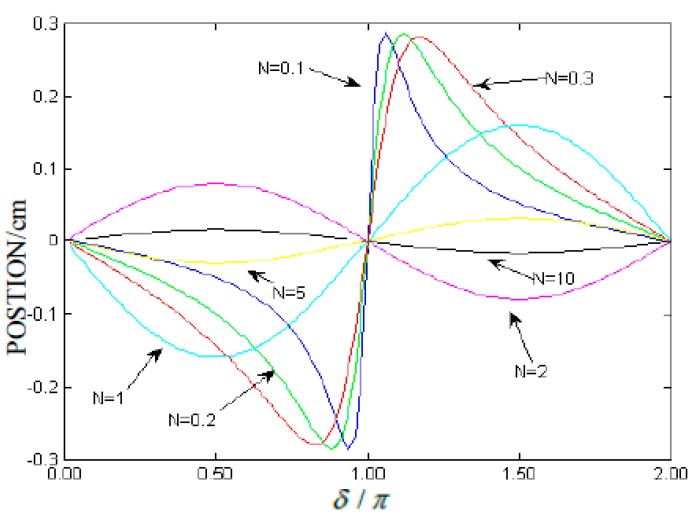
PSD readings *versus* interference fringes phase difference for some values of N.

The simulation curve of Equation (11) is given in Figure 3. Comparing Figure 3 with the variation of the centroid position of the interference fringes at some values of N (Figure 1), a significant conclusion can be obtained. When the PSD signal lights are interference fringes, the PSD output is still the interference fringe centroid position, which is similarly closely in line with the PSD-based method to detect the interference fringes. Finally, the change of the interference arm can be carried out directly by analyzing variations of the interference fringe centroid position, and the interference fringe centroid detection will have potential application value. However, there is a small difference between Figure 1 and Figure 3, which is caused by their different simulation algorithms.

## 3. Experiments and Results

**Figure 4:**
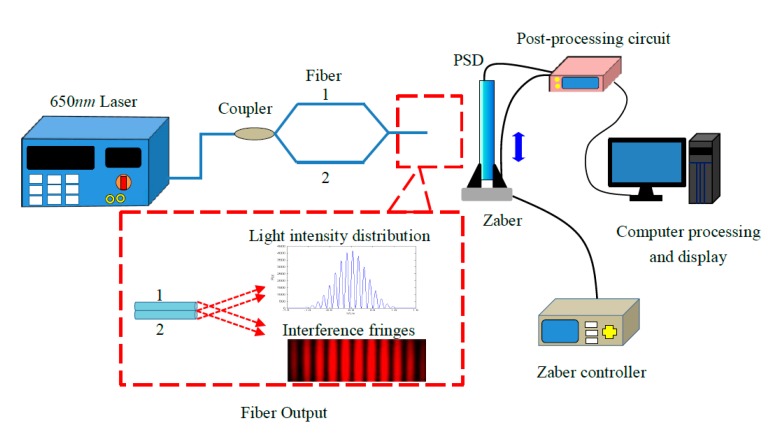
Experimental block diagram of measurement of fringes of displacement based on a single-mode fiber M-Z Space interferometer.

Figure 4 is the experimental block diagram of measurement of displacement fringes based on a SMF M-Z space interferometer. The system consists of a laser light source, 3 dB coupler, PSD detectors, high-precision micro-displacement control platform and a post-processing circuit.

The operating principle of the experimental system is described as follows: the output signal from a 650 nm laser is 1 mW, which is split into two signal beams when it passes through a 3 dB coupler. Two single mode fiber (SMF) output ends for transmitting are tightly arranged and enclosed in a capillary glass tube, and the distance between the two fiber cores is 125 μm. Then, the two beams can be considered as two point light sources and interfere with each other at the output fiber end. When the interference fringes lie on the photosensitive surface of the PSD, the output of the PSD is demodulated by a post-processing circuit. In addition, relative movement of the interference fringes can be measured by the high-precision micro-displacement control platform. The movement of the interference fringes can be obtained by the changes of the two displayed results.

### 3.1. Device Calibration

The PSD (GD3191Z, Associated Opto-electronics Corp., Chongqing, China) used in the experiment is a kind of Si-PIN one-dimensional PSD. The photosensitive surface size is 2 mm × 15 mm, and its resolution is 1 μm, with a responsivity of 0.55 μA/μW*@*0.94 μm.

**Figure 5:**
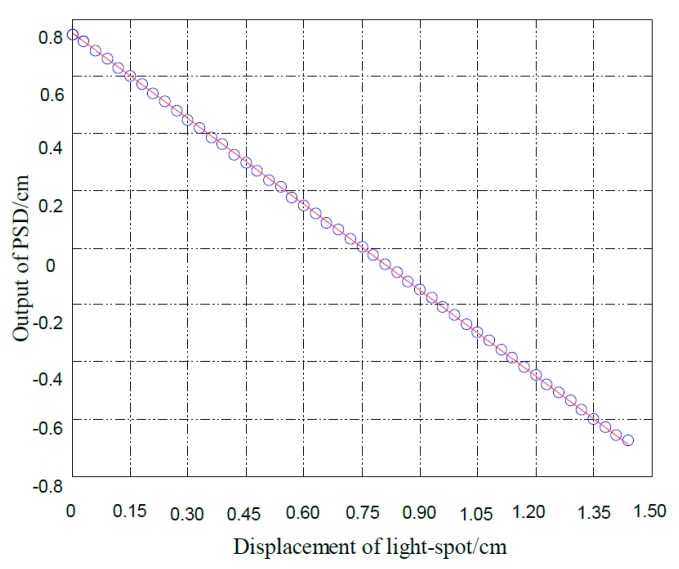
The calibration curve of the PSD.

**Figure 6:**
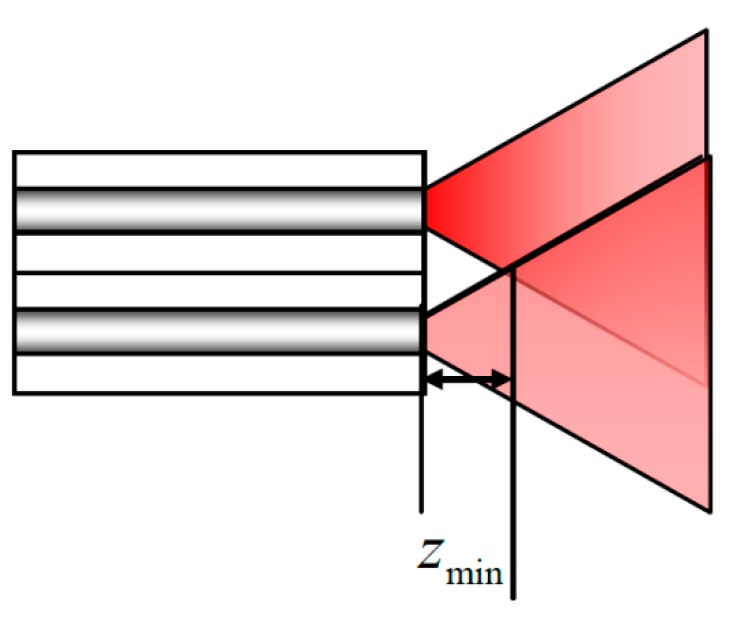
Minimum distance between optical fiber ends and observation plane.

Because a PSD is a semiconductor position sensitive device based on a lateral spot effect, there may be a non-linear response in the measurement. We conducted a single calibration on the PSD, the calibration curve is given in Figure 5. It is obvious that the PSD has a good linearity, and can meet the test requirements. The interference fringes can be observed when two beams from the fiber are overlapping with each other, so we should ensure that the viewing plane is in the beam overlap region, *i.e.*, the distance between the fiber ends and the viewing plane should meet certain requirements. A schematic of the minimum distance between the optical fiber ends and viewing plane is shown in Figure 6.

In the Figure 6, a condition must be met for the distance *z* between the output fiber end and the viewing plane is that the radius of the light spot from a SMF is equal to the distance between the two optical fiber cores. The distance *z* between optical fiber ends and viewing plane is given by Equation (12):

(12) $$l=\text{ω}(z)={\text{ω}}_{0}\sqrt{1+{\left(\frac{{\text{λ}}_{0}z}{{\text{πω}}_{0}^{2}n}\right)}^{2}}$$

where, *l* is the distance between the fiber cores, ω(*z*) is the radius of output optical mode field, ${\text{ω}}_{0}$, ${\text{λ}}_{0}$, n are the Gaussian waist radius of the light field, center wavelength and refractive index of the optical fiber, respectively. In this measurement system, the minimum distance between optical fiber ends and observation plane is *z*_min_ ≈ 6.4 mm. In addition, the stability of the measurement is tested in a thermostatic laboratory by a power meter, and the fluctuation of the output optical power is within 2 μW.

### 3.2. Results and Discussion

**Figure 7:**
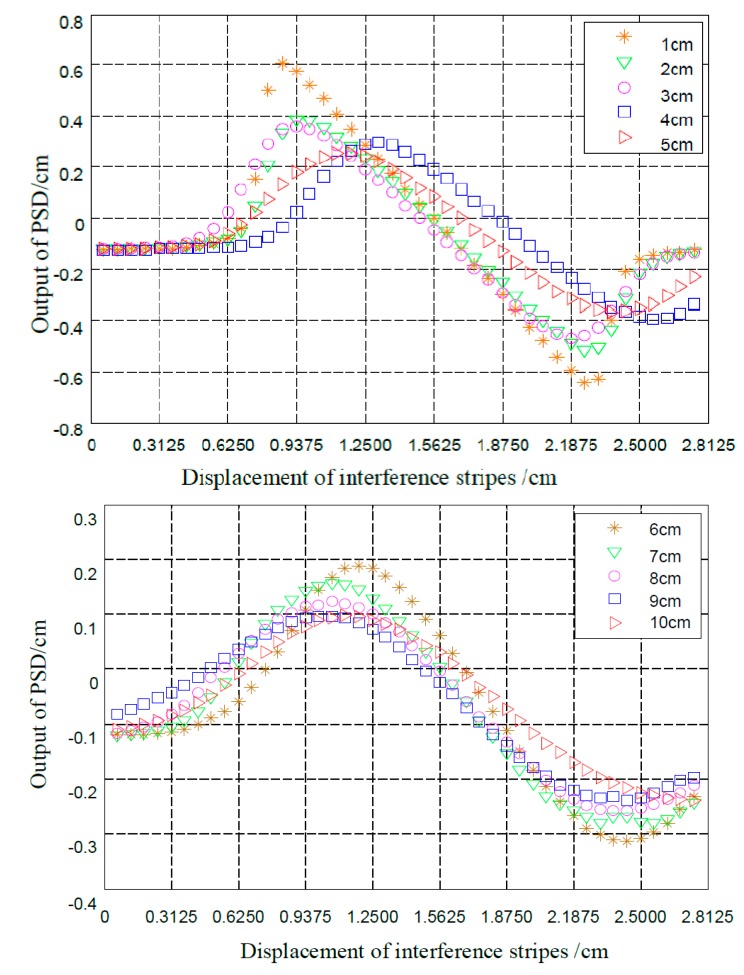
The characteristic curves of the PSD.

In this paper, an experimental one-dimensional PSD moving interference fringes detection system was implemented, based on the centroid interference fringe method, and numerous experiments were conducted. We tested the movement of interference fringes under different conditions and the minimum resolution of the system. With the displacement platform moving in the forward direction, the characteristic curve of the PSD output under different operating distances and the movement of interference fringes is shown in Figure 7, where the slope of curves at different distances between the fiber end and PSD were obtained and exhibited perfect in smoothness and regularity. Particularly, the curve of the distance *z* = 1 cm has an area with very high slope, which illustrates that it has a better PSD output resolution compared with the other distances. Within the linear region of *z* = 1 cm, the fitting result of PSD output *y* and the movement of the interference fringes is given as *y* = −0.9396*x* + 1.4599 (with the degree of fitting of *R*^2^ = 99.98% and the standard deviation of RMSE = 0.009445). When the interference fringes move to 1 cm, the PSD output is aligned to 0.9396 cm. In addition, as the signal light is an interference fringe, a phase difference is bound to exist in the measurement and this affects positioning accuracy.

**Figure 8:**
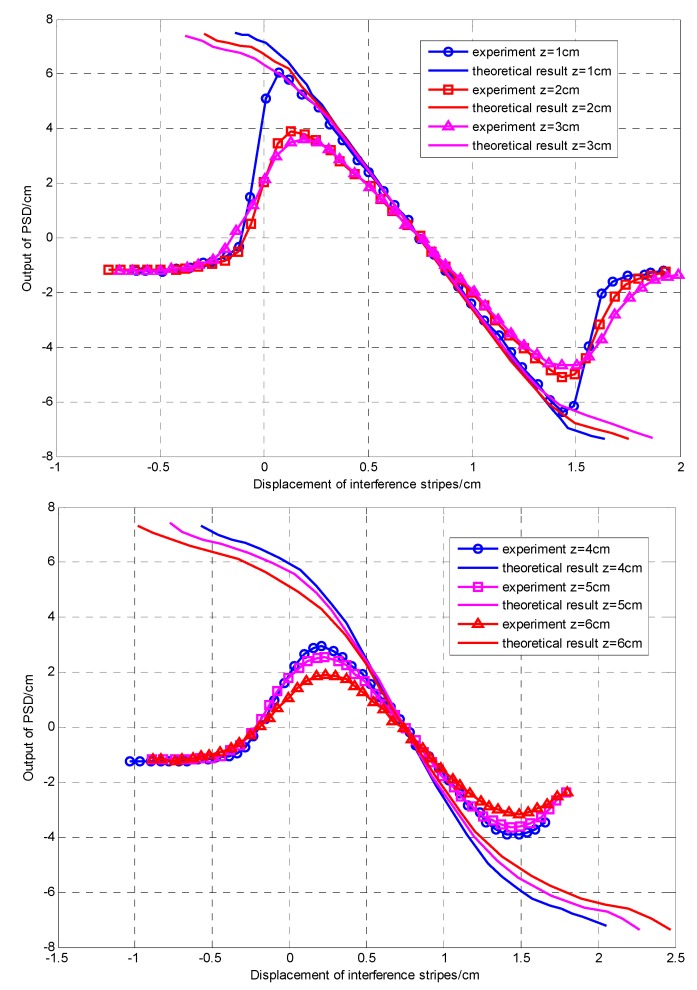
The characteristic curves of the PSD.

The experimental curves and theoretical curve with the distance between the fiber end and the viewing screen from *z* = 1 to 6 cm as the viewing screen moves up from the bottom to observe the centroid position of the interference fringes, are shown in Figure 8 (*λ*_0_ = 632.8 nm, 2*l* = 125 µm) initial phase difference π/2). According to [22], the interference field light intensity of a M-Z interferometer in the viewing screen is given by Equation (13):

(13) $$I(x,y,z)=\frac{A_{0}}{w^{2}(z)}\exp\left(-\frac{2(x^{2}+y^{2}+l^{2})}{w^{2}(z)}\right)\left\{\begin{array}{l} \exp\left(-\frac{4xl}{w^{2}(z)}\right)+\exp\left(\frac{4xl}{w^{2}(z)}\right)+\\ 2\cos\left(\frac{2klx}{R(z)}-\phi \Delta (z)\right) \end{array}\right\}$$

where, A_0_ is the center amplitude of the Gaussian beam, *R*(*z*) is the equiphase surface curvature radius of Gaussian beam, and *x*, *y* are the coordinates of the viewing screen. The theoretical curve is obtained from Equations (3) and (13).

As presented in Figure 8, the first and third parts of all the curves are nonlinear, which is caused by the characteristics of the interference light intensity distribution. The nonlinearity character that exists in the second part is due to the fact that the length of the viewing screen is smaller than the range of the interference fringes. In actual engineering, we need to avoid this kind of nonlinearity. From [23], a conclusion can be obtained that the smaller the distance *z* is, the smaller the interference fringe spacing is, but the number of interference fringes remains the same within the scope of observation. Therefore, we should select an appropriate value of *z* to reduce the scope of the nonlinear part.

A close look at Figure 8 shows that the smaller the working distance *z* is, the better the consistency between the experimental and theoretical curve, because the light source and the diameter of the interference field affect the position precision of the PSD. Moreover, due to the fabrication of the PSD, the uneven or hopping resistivity distribution on PSD photosensitive surface also can lead to inaccurate positioning at the PSD edges. In the interference field with a large diameter, most of the light spots are distributed in the area of low precision, which makes the theoretical curve inconsistent against the experimental curve. At a larger interference field, we can sort out the numerical tables corresponding to the relationship between the experimental and theoretical curve, and they can be applied in measuring the accurate position of the interference fringes.

**Figure 9:**
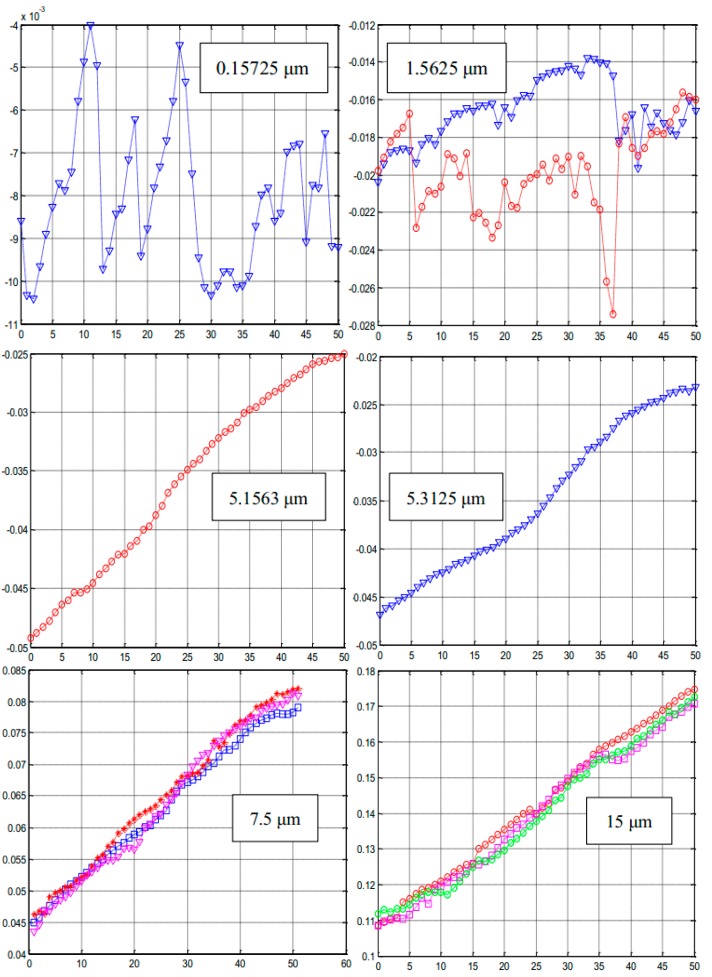
The characteristic curves of the PSD.

In order to analyze the minimum resolution of the setup shown in Figure 4, we set the interval of the displacement platform (the relative movement of the interference fringes) as 0.15725, 1.5625, 5.1563, 5.3125, 7.5 and 15 μm, respectively. The characteristic curves of PSD output under different movement of interference fringes are shown in Figure 9 (the *x* coordinate is measuring time/s, *y* coordinate is the PSD output/mm). It can be clearly concluded that the best resolution of the system is seen at 5.1625 μm with the working distance set by *z* = 1 cm, during the measuring time of 50 s, the PSD output range is 0.025 mm, and the curve also shows the best linearity.

### 3.3. Setup Analysis and Evaluation

**Figure 10:**
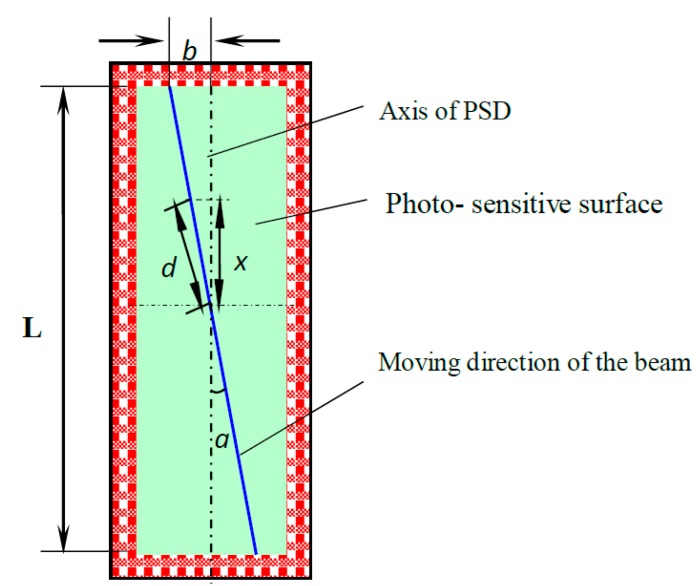
Beam positions on the PSD’s photosensitive surface.

In our experiments, the devices and instruments in the system always have some errors, for example, when the axis of the PSD is deviated from the moving direction of the beam, the light spot moves up and down as the interference fringes change. As shown in Figure 10, the size of the PSD light-sensitive surface is 1 mm × 15 mm, and maximum deflection between the light spot position and PSD axis is 2*b =* 0.5 mm, the maximum error Δx between the actual displacement *d* and the measured axial displacement *x* yields the relative error. The expression for the relative error is given by Equation (14):

(14) $$\frac{\Delta x}{d}=\frac{d-x}{d}=\frac{d-d\cos\text{α}}{d}=1-\cos\text{α}=1-\frac{L/2}{\sqrt{{\left(L/2\right)}^{2}+b^{2}}}$$

In specific experimental parameters, we calculate that the relative error is about 0.12%, which can be eliminated through the calibration of the system.

The other errors existing in the system are summarized below: the electrode dark current and stray light existing in the PSD yield an output error of about 0.5%, and the interference fringe position measurement error caused by it is ΔS_1_ = 0.21‰. In addition, the light intensity varies within the range of microwatt magnitude and has a 0.5% of PSD output error, which also causes an interference fringe position measurement error of ΔS_1_ = 0.21‰.

The PSD is susceptible to be disturbed in the experiment by the background light, which often affects the measurement accuracy and reliability. This paper firstly proposed a preliminary attempt to overcome this defect with a one-dimensional photonic crystal structure. There is a narrow transmission window in the photonic band gap of photonic crystals. The 3 dB bandwidth of the window at the wavelength of 650 nm is 1 nm, whose transmittance is close to 100%. This kind of structure can fundamentally solve the problem of background light influence on the PSD.

## 4. Conclusions

This paper designs a set of one-dimensional PSD systems to detect the movement of the interference fringes in a M-Z interferometer based on the interference fringe centroid method. By performing measurements of the minimum operating distance and the PSD output curves in the measurement system, a best resolution of the whole system of 5.1563 μm can be obtained. Finally, we analyze the errors of the devices and instruments in the system, and assess their effects on measuring the interference fringes’ position. Our approach is still a preliminary attempt to detect interference fringes using the PSD, and some factors in specific applications have not been taken in to account, such as the interference in the light paths. In order to overcome the background light in the experiment, an ultra-narrow-band filter based on the one-dimensional photonic crystal is suggested. Further studies should focus on optimizing the measurement system structure, but a measurement system with perfect stability and high sensitivity has potential application prospects.
